# Real-world evidence evaluation of LDL-C in hospitalized patients: a population-based observational study in the timeframe 2021–2022

**DOI:** 10.1186/s12944-024-02221-x

**Published:** 2024-07-24

**Authors:** Umberto Capece, Chiara Iacomini, Teresa Mezza, Alfredo Cesario, Carlotta Masciocchi, Stefano Patarnello, Andrea Giaccari, Nicoletta Di Giorgi

**Affiliations:** 1 Centro Malattie Endocrine e Metaboliche, Dipartimento di Scienze Mediche e Chirurgiche, Fondazione Policlinico Universitario Agostino Gemelli IRCCS, Rome, Italy; 2 Dipartimento di Medicina e Chirurgia Traslazionale, Università Cattolica del Sacro Cuore, Rome, Italy; 3 Real World Data Facility, Gemelli Generator, Fondazione Policlinico Universitario Agostino Gemelli IRCCS, Rome, Italy; 4 Pancreas Unit, Medicina Interna e Gastroenterologia, CEMAD Centro Malattie dell’Apparato Digerente, Fondazione Policlinico Universitario Agostino Gemelli IRCCS, Rome, Italy; 5 Gemelli Digital Medicine & Health, Rome, Italy; 6 Open Innovation Unit, Scientific Directorate, Fondazione Policlinico Universitario A. Gemelli IRCCS, Rome, Italy

**Keywords:** Real-world evidence, Cardiovascular risk, Frail, Inpatients, LDL cholesterol, Lipid-lowering therapies

## Abstract

**Aims:**

European registries and retrospective cohort studies have highlighted the failure to achieve low-density lipoprotein-cholesterol (LDL-C) targets in many very high-risk patients. Hospitalized patients are often frail, and frailty is associated with all-cause and cardiovascular mortality. The aim of this study is to evaluate LDL-C levels in a real-world inpatient setting, identifying cardiovascular risk categories and highlighting treatment gaps in the implementation of LDL-C management.

**Methods:**

This retrospective, observational study included all adult patients admitted to an Italian hospital between 2021 and 2022 with available LDL-C values during hospitalization. Disease-related real-world data were collected from Hospital Information System using automated data extraction strategies and through the implementation of a patient-centered data repository (the Dyslipidemia Data Mart). We performed assessment of cardiovascular risk profiles, LDL-C target achievement according to the 2019 ESC/EAS guidelines, and use of lipid-lowering therapies (LLT).

**Results:**

13,834 patients were included: 17.15%, 13.72%, 16.82% and 49.76% were low (L), moderate (M), high (H) and very high-risk (VH) patients, respectively. The percentage of on-target patients was progressively lower towards the worst categories (78.79% in L, 58.38% in M, 33.3% in H and 21.37% in VH). Among LLT treated patients, 28.48% were on-target in VH category, 47.60% in H, 69.12% in M and 68.47% in L. We also analyzed the impact of monotherapies and combination therapies on target achievement.

**Conclusions:**

We found relevant gaps in LDL-C management in the population of inpatients, especially in the VH category. Future efforts should be aimed at reducing cardiovascular risk in these subjects.

**Supplementary Information:**

The online version contains supplementary material available at 10.1186/s12944-024-02221-x.

## Introduction

The 2021 ESC report confirms that cardiovascular disease is still the main cause of death in Europe, even though the number of cancer deaths in some countries exceeds the number of cardiovascular disease (CVD) deaths [1]. The burden of dyslipidaemia has increased over the past 30 years as high LDL cholesterol (LDL- C) was considered the 15th leading risk factor for death in 1990, rising to 11th place in 2007 and 8th in 2019 [2]. However, given the possibility of highly effective interventions, dyslipidaemia is a modifiable risk factor [3]. The 2019 update to the European Society of Cardiology/European Atherosclerosis Society (ESC/EAS) guidelines for lipid management, defines different LDL-C targets for different cardiovascular risk categories (< 116 mg/dL for low risk, < 100 mg/dL for moderate risk, < 70 mg/dL for high risk and < 55 mg/dL for very high risk) [4].

European data from registries and retrospective cohorts have shown that very high cardiovascular risk patients do not achieve guideline-recommended LDL-C targets [5]. Similarly, a consistent number of off-target patients has been found in other highly selected populations [6, 7]. However, an evaluation of this relevant issue in hospitalized patients is still lacking. While hospitalization is a feared consequence of several diseases and their combination, it allows for continuous medical assistance and access to advanced care. Hospitalized patients are usually frailer [8] which is associated with higher all-cause in-hospital mortality [9] and increased mortality after acute coronary syndromes [10]. Studies have found a significant association between frailty and both all-cause mortality and cause-specific mortality, such as ischemic heart disease and cerebrovascular disease [11]. Further, hospitalized patients also have a greater occurrence of comorbidities, which is an independent predictor of negative cardiovascular outcomes after hospital discharge [12]. Therefore, it is imperative to develop comprehensive risk reduction strategies for hospitalized patients.

The aim of this study was to evaluate LDL-C in patients from the general population, admitted to the Fondazione Policlinico Agostino Gemelli IRCCS Hospital (FPG), in order to classify patients into different cardiovascular risk categories and highlight treatment gaps in LDL-C management.

## Methods

### Study design and ethical approval

This retrospective, observational, cross-sectional study included hospitalized patients with at least one of the following measurements available during hospitalization: total cholesterol, LDL-C, high-density lipoprotein cholesterol (HDL-C) and triglycerides (TG). The patient recruitment period lasted from 1st January 2021 to 31st August 2022. Baseline data were collected using data extraction strategies. No ad hoc visits, examinations, laboratory tests or procedures were mandated as part of this non-interventional observational study.

The study protocol conforms to the ethical guidelines of the 1975 Helsinki Declaration and was approved by the Ethics Committee of the Fondazione Policlinico Gemelli Hospital (prot. no. 16,832/23). The study used anonymous data and, according to national and European regulations, a waiver was applied to the requirement for patient informed consent (further details on *ethical aspects* are reported in paragraph *2.3.6 Ethical aspects*).

### Patient population

**Fig. 1:**
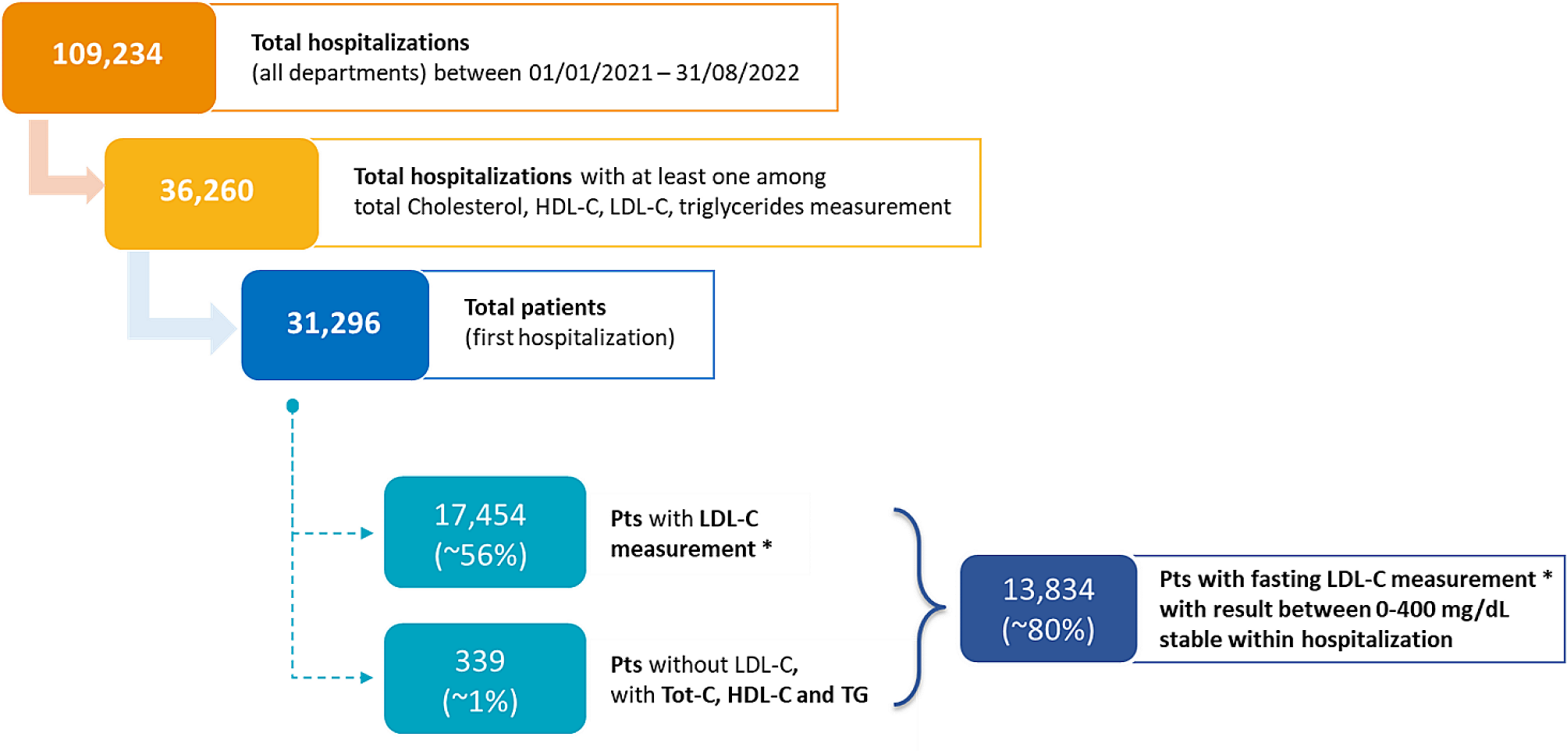
Flow chart of the study. *All LDL-C measurements were assessed using Friedewald formula [13]

The study population consisted of patients aged over 18, hospitalized at FPG and with at least one lipid profile measurement available (total cholesterol/LDL-C/HDL-C/TG). Patient selection and study flow are shown in Fig. 1. Baseline was defined as the first hospital admission per patient and laboratory results were abstracted from the hospital Laboratory Information System (LIS). Cholesterol and triglyceride measurements were retrieved only if conducted at least 36 h after artificial nutrition and/or interfering (non-lipid-lowering) drugs (80% of 60,292 total measurements, nutritional substances are listed in Supplementary Table S1). If no LDL-C was available, and total cholesterol, HDL-C and TG were reported, LDL-C levels were calculated with the Friedewald formula [13]. Individuals were excluded if: (1) lipid results were missing; (2) LDL-C could not be calculated (i.e. triglycerides were > 400 mg/dL, hence Friedewald formula could not be used); (3) LDL-C calculated < 0 mg/dL or > 400 mg/dL; or (5) LDL-C results were not stable (delta min-max/mean > 30%). For each patient all LDL-C measurements retrieved during the first hospitalization in the inclusion period were considered and, if the measurement was stable (delta min-max/mean < 30%), the mean LDL-C value was attributed to the patient as baseline LDL-C level.

### Data extraction methodology

**Fig. 2:**
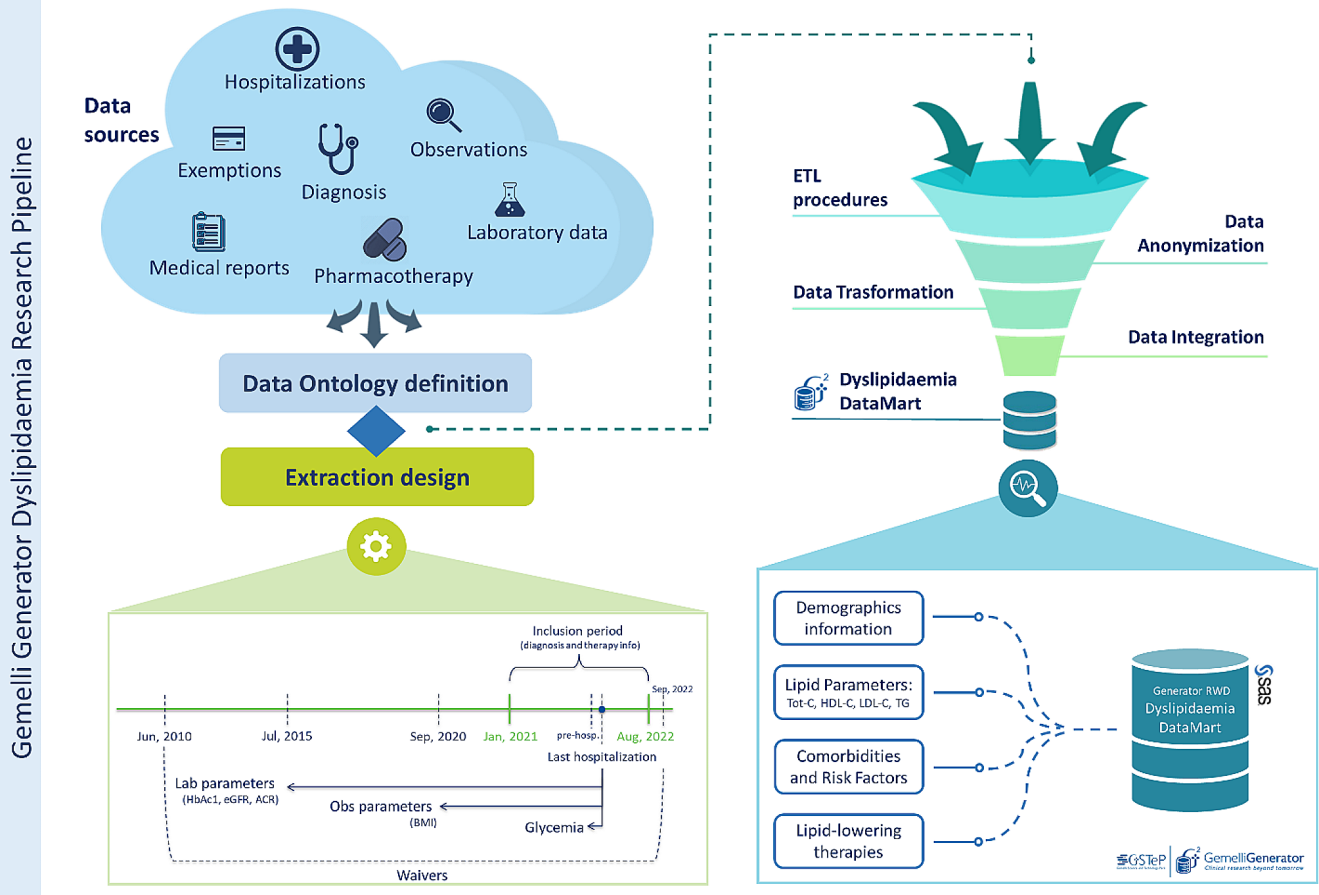
Overview of Gemelli Generator Dyslipidaemia research pipeline. ETL: Extract: Transform: Load; BMI: Body mass index; Tot-C: total cholesterol; HDL-C: high-density lipoprotein cholesterol; LDL-C: low-density lipoprotein cholesterol; TG: triglycerides

Data extraction was based on a general methodology to generate real-world evidence (RWE) used by the Generator Real World Data (RWD) research facility, created at FPG to support clinical staff in data-driven research activities [14]. The methodology is based on the design and implementation of a patient-centered data repository (the Dyslipidaemia Data Mart) that extracts, validates, and integrates all relevant data sources for patient characterization and risk assessment based on current/ target LDL-C levels. The construction of the Data Mart was based on the Data Dictionary (Supplementary Table S2) defined by an interdisciplinary team of endocrinologists and diabetologists, who identified an extensive list of disease-related variables to be considered for the study. The patient-centered Data Mart includes a subset of data from the hospital’s information technology (IT) warehouse, about the specific dyslipidemic domain: specifically, it collects demographic information, hospitalization information, laboratory and clinical parameters, comorbidities, risk factors and ongoing therapies (Fig. 2). For each subset, available data sources within data warehouse (DWH) are detected and investigated by data analysts. Specifically, structured and unstructured data sources that provide variables of interest are identified within the Hospital Information Systems (HIS), as detailed in Supplementary Table S2. Structured data is characterized by a high level of standardization and encoding, fulfilling specific ontologies and common data formats (e.g. code for diagnosis according to the International Classification of Diseases, 9th revision - Clinical Modification, ICD9-CM). This type of data is collected without further processing. Conversely, unstructured data refer to information from medical reports in free text format, which require further processing for subsequent analysis. The extraction procedure design is then constructed also in consideration of the time range of interest for different parameters, established according to their clinical significance.

Specific ETL (Extract, Transform, Load) procedures are implemented to automatically retrieve data from heterogeneous data sources: direct extraction from structured data sources and clinically validated text mining techniques from unstructured data sources.

At the end of the data extraction workflow, data are pseudonymized, through industry-standard algorithms to encrypt sensitive patient data (such as Patient ID and Admission ID) and fulfill privacy requirements. Finally, data transformation and integration are performed to store data in dedicated tables, grouped by data source and/or data category and indexed using pseudonymized patient IDs. Data validation is performed during the entire workflow, mainly through standardized reporting processes.

The technological tools used are SAS^®^ v. 9.04 and R v. 4.2.1, which are among the most common programming environments in data science. SAS^®^ is used as a middleware for ETL tasks from HIS, as a data repository including tables in dedicated storage areas (SAS VIYA Caslibs), and as a text mining tool for extracting clinical concepts. R is used for data analytics, data processing, data visualization and modeling activities.

#### Hospitalizations

Based on the matching criteria for inclusion of a subject in the retrospective cohort, all inpatient electronic health records (EHR) were automatically extracted from the hospital systems. Hospital admission, demographic information (age at hospitalization, gender), data on diagnosis contained in discharge reports and procedures conducted during hospitalization were retrieved. The first hospitalization per patient within the inclusion period (1st January 2021–31st August 2022) was considered as the baseline.

#### Lipid measurements

Lipid measurements (total cholesterol, LDL-C, HDL-C, TG) were automatically extracted from the LIS when diagnostics were performed at hospital facilities during hospitalization.

Both technical and clinical quality controls were conducted on the data to ensure consistency of the extracted lipid measurements. Acceptable values for each variable were assessed and time distance from artificial nutrition was considered to ensure that the measurement was performed at fasting status. For LDL-C measurements with results calculated from LIS data, acceptable criteria for the application of the Friedewald formula were re-assessed. All values without fulfilled requirements (i.e. measurements with TG > 400 mg/dL) were excluded to avoid underestimation of LDL-C. For LDL-C measurements, a validation scheme using evidence from different data sources was implemented under the guidance of clinicians, who also conducted independent checks on the quality of the results, mainly involving patients with LDL-C measurements near the upper limits of range acceptability (i.e. LDL-C > 250 mg/dL). Finally, the trend in LDL-C measurements was assessed and patients with high variability in results were excluded from the study population.

#### Comorbidities and risk factors

Multiple data sources were integrated, as reported in Supplementary Table S4, to define comorbidity status for patients. All available data were collected for each comorbidity, (availability assessment for each source is detailed in Supplementary Table S5) including:

- Structured sources:
  - primary and secondary diagnosis reported during hospitalization (ICD9-CM at discharge);
  - payment exemption data extracted from the outpatient data that DWH provides to the national healthcare system (time range: Jun 2010 – Sep 2022);
  - laboratory and observational parameters extracted from hospital LIS and EHR (Observations), respectively (time range: before hospitalization discharge date per patient or between specific intervals during the hospitalization).
- Unstructured sources:
  - information extracted from clinical reports during hospitalization on therapies using drug name/active ingredient/ATC codes, if specific treatments are available for the pathological condition.

Data to assess three main comorbidities for dyslipidaemic patients, diabetes, chronic kidney disease (CKD) and obesity, were extracted. Due to length limitations, we will only detail the case of diabetes. Patients were considered as diabetics if meeting at least one of the following: ICD9-CM codes 250.*; payment exemption codes 013 and 013.250; on anti-diabetic therapy (152 drugs/51 ATC detailed in Supplementary Table S6, excluding metformin) extracted from hospitalization clinical reports (clinical diaries, medical histories, discharge letters); glycated hemoglobin (HbA1c) ≥ 6.5% before discharge date; or mean fasting blood glucose ≥ 126 mg/dL for measurements performed between pre-hospitalization/admission and the 5th day from admission. In order to select only blood glucose measurements in fasting status, results were retrieved only if performed together with lipid profile measurements and conducted at least 36 h after medications/nutritional administration. Data integration to determine CKD, obesity and ASCVD is reported in table S4. Concordance assessment for the different variables involved in the definition of comorbidities was performed. Co-occurrence matrices were constructed to assess paired data sources and overlaps between multiple sources were investigated through upset plot and Venn diagram. For example, to better characterize diabetes evidence, each source (ICD9-CM, exemption, therapy, HbA1c and glycaemia) was compared in pairs with the others and intersections between multiple sources were evaluated. Most of diabetic patients (65%) were identified through a combination of two or more evidence sources, others were identified exclusively through laboratory evidence (HbA1c or glycaemia, 30%) or anti-diabetic therapy (5%). Subsequent validation, performed on single evidence test cases with the guidance of clinicians permitted the creation of an identification method (as reported in Supplementary Table S4) to assign comorbidities with a high degree of accuracy.

Relevant risk factors, such as systolic/diastolic blood pressure (SBP/DBP) and smoking status, were retrieved from data collected in HIS during hospitalization. Specifically, SBP/DPB measurements were extracted from EHR Observations in the form of structured data, while smoking status was collected from medical history acquired at admission through both structured and unstructured data (i.e. medical notes written in free text format) available in HIS. Text mining algorithm techniques were performed to process medical history sections and extract the clinically relevant variable related to smoking status. The different data sources were analyzed concurrently so as to consider unstructured data only if structured data were unavailable.

#### Lipid-lowering therapies

First, a reference drug database including all relevant lipid-lowering therapies (LLT), mainly statins, ezetimibe and proprotein convertase subtilisin/kexin type 9-inhibitors (PCSK9-i) in mono or combination therapy, was constructed in collaboration with the clinical team (Supplementary Table S7). Information on LLT before the baseline was extracted from inpatient EHR: data on therapies before admission and administration during hospitalization with relative dosages were retrieved as structured data. Specifically, therapy before admission was considered and, when not available, it was integrated with data on the first administration during hospitalization. Finally, if neither of these two different data sources were available, information on LLT was extracted as drug name/active ingredient/ATC code from clinical reports collected during hospitalization (clinical diaries, medical histories, discharge letters). According to details on treatment dosage, statin therapies were defined as low, medium and high-intensity (LI-statin, MI-statin and HI-statin) based on statin molecules and daily dose, according to Hodkinson et al. [15]. When no data on treatment dosage were present (i.e., data extracted from plain text) statin therapies were defined as ND-statins.

Validation of LLT extracted from reports improved text mining rules through distance-based rules established to filter expressions referring to immune reactions (drug allergies and/or intolerances), treatment discontinuation or therapy interruptions. Finally, concordance for different LLT data sources was assessed for those cases in which data from different sources were effectively available, to examine coherence of therapy evidence as an index of data consistency. For this sub-population co-occurrence matrices for different LLT therapies were constructed comparing LTT therapy before admission and first LLT administration during hospitalization.

#### Ethical aspects

All privacy issues were analyzed with the Policlinico Gemelli’s Data Protection Officer, to design an approach fully compliant with Italian and European GDPR directives and regulations (EU Directive 2016/679 and Italian Laws: Decreto Legislativo 196/2003, Decreto Legislativo 101/2018, Autorizzazione Generale Garante 9/2016). These principles of Ethics and Governance are clearly stated in a legally relevant public document, the Generator Real World Data Facility Umbrella Protocol.

### Definition of risk categories and LDL targets

Cardiovascular disease (CVD) risk categories were defined according to the 2019 ESC/EAS Guidelines on dyslipidemia [4] as low (L), moderate (M), high (H) and very high (VH) risk. CV risk category for each patient at baseline was assessed using information on patient demographics, comorbidities, risk factors and Systematic COronary Risk Evaluation (SCORE). SCORE calculation to estimate 10-year risk for fatal cardiovascular disease (coronary heart disease and non-coronary disease) was performed on the basis of age, gender, smoking status, total cholesterol and SBP, according to Conroy et al [16], for all patients excluding those already defined as VH CVD risk patients (25.9% of the entire study population) since no risk estimation models are needed for such patients and they all need active management of all risk factors. Smoking was coded as 1 for current and 0 for non-smoker and when smoking status was not available (36.7% of the entire study population) multiple imputation by chained equations (MICE) technique was used to improve the quality of smoking records [17]. The imputation was conducted using all the remaining parameters needed for SCORE calculation (sex, age, total cholesterol, systolic pressure) and the LDL-C parameter, to maintain consistency with the overall SCORE distribution. MICE imputation was performed through *logreg* function and a total number of 100 imputations were performed and subsequently integrated so that if *p*(1) > 0.5 then smoking status was considered as 1, otherwise as 0. Total cholesterol and SBP were defined as mean value for total cholesterol (in mmol/L) and SBP (in mmHg) measurements, respectively, collected during baseline hospitalization.

For CVD risk category assessment, patients with type-1 diabetes mellitus were defined by all diagnoses including juvenile onset diabetes (ICD9-CM codes 250.01|250.03|250.11|250.13|250.41|250.71|250.73|250.91), while diabetic patients with target organ damage were defined by diagnosis of complicated diabetes (ICD9-CM codes 250.4|250.5|250.6|250.7|250.8|250.9) or evidences of albuminuria (presence of at least one measurement outside normal range within +/- 90 days from the baseline hospitalization). To estimate diabetes duration, we considered the average age of onset for the Italian population according to annual reports: for diabetic patients ≥ 70 years type-2 diabetes mellitus duration was defined as ≥ 10 years; for type-1 diabetic patients > 45 years type-1 diabetes mellitus of long duration was defined as > 20 years. Familial hypercholesterolemia (FH) was assessed using exemption code RCG070.

The same guidelines were used to define LDL-C targets in each of ESC/EAS CVD risk categories. Patients were considered on target if their baseline LDL-C level was below the target LDL-C, as suggested by 2019 ESC/EAS Guidelines, according to their CVD risk category. Also, to have a measure of the mismatch for LDL-C goal achievement, a continuous evaluation based on the distance to target (DTT) between current LDL-C levels and the recommended LDL-target was conducted.

### Statistical analysis

Clinical characteristics are presented as means ± standard deviations (SD) for continuous variables and as numbers and relative percentages for categorical variables. Results are reported by CV risk classification (calculated using patient data and applying the CV risk classification of 2019 ESC/EAS), cholesterol targeting LLT, and by proportion of patients achieving LDL-C goals (on-, off-target).

Continuous variables were compared between groups using one-way ANOVA test. Otherwise, categorical variables were compared between groups using Chi-square test. All statistical tests were two sided and were performed at the 5% level of significance, unless otherwise stated. Data were analyzed using R software (version 4.2.1).

## Results

### Clinical demographics and patient characteristics

The extraction process retrieved 109,234 hospitalizations from all FPG departments between 01/01/2021 and 01/08/2022. After a selection process (Fig. 1), we identified 13,834 admitted patients with available fasting LDL-C measurement between 0 and 400 mg/dL which was stable during hospitalization. The average age of the population was 65.2 ± 16.5 years, 57.2% were males, and the average LDL-C value was 87.4 ± 34.6 mg/dL. More than half the patients (67.3%) had LDL-C value higher than 70 mg/dL. ASCVD prevalence was 31.8%, while 30.5% patients had a diagnosis of diabetes and 35.7% of CKD. Several patients were affected by more than one comorbidity, including diabetes, CKD, and obesity (17.9% had 2 comorbidities, 3.6% had 3 comorbidities). The most prevalent ICD9 codes in primary diagnoses were: 480.41 (Covid-19 pneumonia), 434.01 (Cerebral Infarction due to thrombosis of unspecified cerebral artery) and 414.01 (Coronary atherosclerosis of native coronary artery). A detailed description of patient characteristics can be found in Supplementary Table S8.

### Cardiovascular risk classification

Combining clinical (ICD9-CM codes, exemption codes, drug therapies) and laboratory data with observational parameters, and data obtained from the SCORE calculation, we classified patients into risk categories. The majority of patients (49.76%) were at very high risk, followed by low risk (17.2%), high (16.2%) and moderate (13.72%). In the very high-risk category ASCVD was present in 63.9% patients, diabetes in 46.5%, CKD in 54.4%, obesity in 18.7% (as reported in Table 1).

**Table 1:** Clinical characteristics and risk factors by CV risk classification according to 2019-ESC/EAS guidelines. Continuous variables are reported as means with relative standard deviation and *p*-values refer to one-way ANOVA test. Categorical variables are reported as numbers and relative percentages and *p*-values refer to Chi-square (χ²) test

	All	Low	Moderate	High	Very High	AOV/Chi2-square test
*N*	13,834	2,372 (17.2%)	1,898 (13.7%)	2,327 (16.8%)	6,884 (49.8%)	
Age (yrs)	**65.2 ± 16.5**	43.7 ± 10.9	63.2 ± 8.6	71.4 ± 12	71.8 ± 13.7	*0*
Males	**7908 (57.2%)**	1031 (43.5%)	1195 (63%)	1197 (51.4%)	4288 (62.3%)	*1.628e-67*
Lipid profile						
LDL-C (mg/dL)	**87.4 ± 34.6**	93.1 ± 30.3	95 ± 30.7	88.8 ± 40.2	82.1 ± 34.2	*2.128e-69*
DTT (mg/dL)	**12.4 ± 39.4**	-22.9 ± 30.3	-5.0 ± 30.7	18.8 ± 40.2	27.1 ± 34.2	*0*
DTT %	**25.7 ± 59.8**	-19.8 ± 26.1	-5 ± 30.7	26.9 ± 57.5	49.3 ± 62.1	*0*
Total Cholesterol (mg/dL)	**150.5 ± 42.8**	158.7 ± 39	159.8 ± 38.5	151.6 ± 48	143.9 ± 42.2	*3.908e-74*
Triglycerides (mg/dL)	**123.2 ± 58.9**	117.6 ± 60.9	119.5 ± 53.1	122.2 ± 58.9	127.3 ± 59.6	*1.965e-13*
HDL-C (mg/dL)	**39 ± 15.2**	42.1 ± 15.9	41 ± 15.5	38.6 ± 15.1	37.1 ± 14.5	*2.371e-51*
Risk Factors						
BMI (kg/m2)	**26.3 ± 5.7**	25.4 ± 6	25.9 ± 4.6	26.5 ± 5.4	26.6 ± 5.7	*2.311e-11*
BP systolic (mmHg)	**122.7 ± 12.9**	116.2 ± 10.3	122.8 ± 11	123.7 ± 12.5	124.8 ± 13.6	*6.294e-137*
BP diastolic (mmHg)	**72 ± 6.6**	71.1 ± 6.6	73.4 ± 6.7	72.1 ± 6.4	71.9 ± 6.5	*7.787e-17*
Current Smokers	**1504 (10.9%)**	164 (8.9%)	172 (11.1%)	161 (8.1%)	877 (12.7%)	*4.257e-34*
Comorbidities						
Diabetes mellitus	**4218 (30.5%)**	53 (2.2%)	280 (14.8%)	671 (28.8%)	3203 (46.5%)	*0*
CKD	**4937 (35.7%)**	12 (0.5%)	6 (0.3%)	1172 (50.4%)	3742 (54.4%)	*0*
Obesity	**2471 (17.9%)**	402 (16.9%)	265 (14%)	480 (20.6%)	1290 (18.7%)	*7.480e-08*
No Comorbidities (among diabetes, CKD, obesity)	**5684 (41.1%)**	1905 (80.3%)	1349 (71.1%)	616 (26.5%)	1511 (21.9%)	*0*
Single Comorbidity	**5172 (37.4%)**	467 (19.7%)	547 (28.8%)	1099 (47.2%)	3009 (43.7%)	*1.880e-126*
2 Comorbidities	**2480 (17.9%)**	0 (0%)	2 (0.1%)	612 (26.3%)	1866 (27.1%)	*8.706e-304*
All 3 Comorbidities	**498 (3.6%)**	0 (0%)	0 (0%)	0 (0%)	498 (7.2%)	*4.410e-107*
ASCVD	**4401 (31.8%)**	0 (0%)	0 (0%)	0 (0%)	4401 (63.9%)	*0*

Abbreviations. AOV: one-way ANOVA test; ASCVD: atherosclerotic cardiovascular disease; BMI: body mass index; BP: blood pressure; CKD: chronic kidney disease; HDL-C: high-density lipoprotein cholesterol; LDL-C: low-density lipoprotein cholesterol

### LDL-C levels and achievement of LDL‑C targets according to ESC/EAS risk categories

**Fig. 3:**
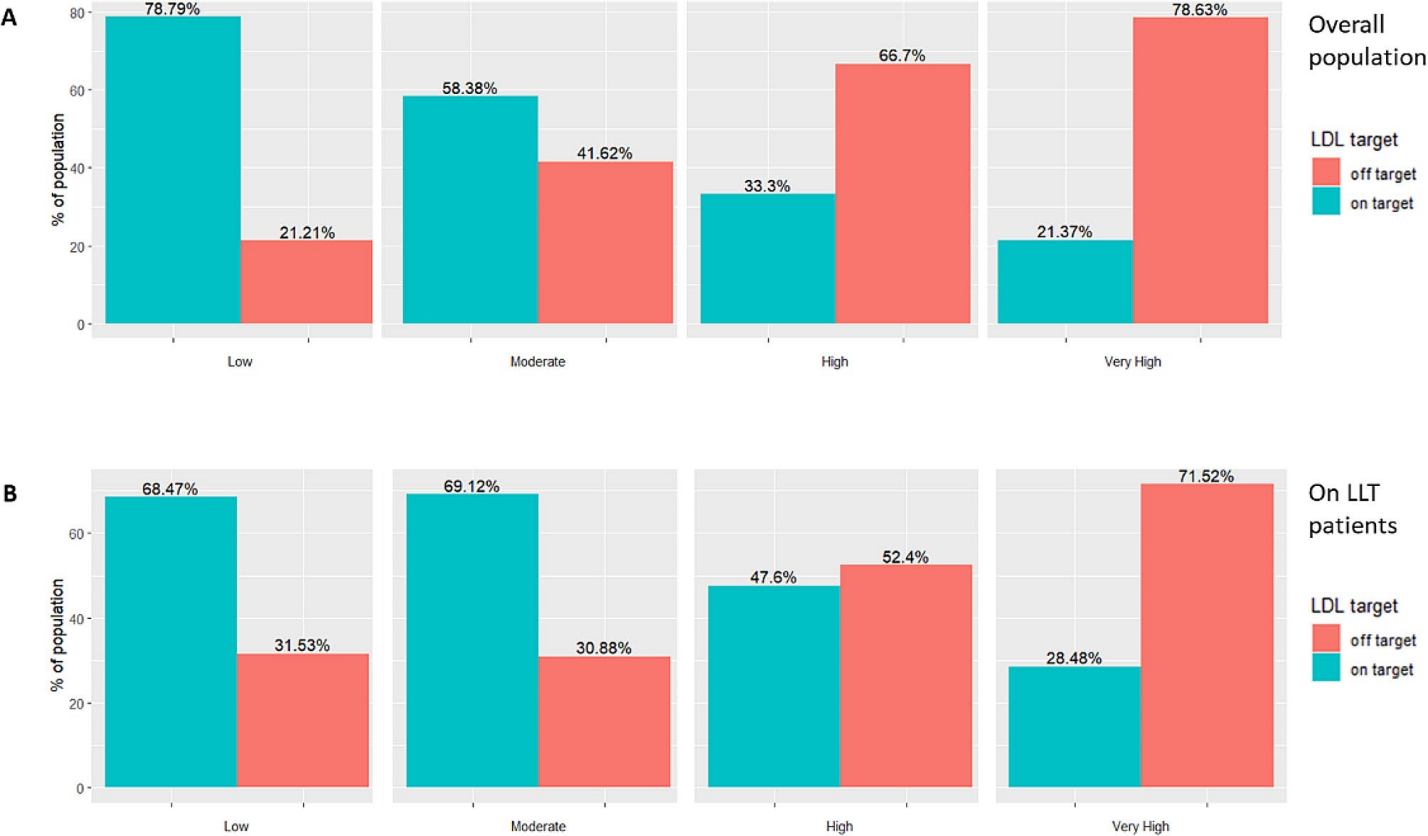
Percentage distribution of on-/off-target patients stratified by CV risk according to 2019-ESC/EAS guidelines (Low, Moderate, High, Very High) for overall population (**A**) and for subgroup of patients on lipid-lowering therapy. (LLT: lipid-lowering therapies) (**B**)

As expected, average LDL-C was significantly and progressively lower, in higher risk categories (Table 1). Conversely, distance to target (DTT) was significantly higher in the very high-risk category compared to the others, both low and moderate risk categories exhibited a negative mean DTT. Similarly, the percentage of on-target patients was progressively lower towards the worst categories (78.8% in low risk, 58.4% in moderate risk, 33.3% in high risk and 21.4% in very high risk) (Fig. 3). Overall, on-target patients were 37.8%, while the percentage of target achievement in specific categories was: 32.1% in diabetes, 28,8% in CKD and 29.1% in hypertension. The overall percentage of on-target patient resulted significantly different in specific sub-groups of patients: lower in elderly ( > = 80 years) patients, and higher in critically ill or cancer patients (as shown in Supplementary Table S9).

### Use of LLT

After the LLT extraction process and integration of therapy data, we identified 4,394 patients with LLT, the remaining number of patients were off-treatment, or the therapy was not retrievable retrospectively. Overall, in our analysis, the percentage of patients on lipid-lowering therapy appeared to be 31.8%. The distribution of LLT in patients was the following: 43% moderate-intensity statins, 35% high intensity statins, 8.77% moderate intensity statins plus ezetimibe, 4.03% high intensity statins plus ezetimibe, 2.98% ezetimibe alone, 2.46% low intensity statins, 1.57% low intensity statins plus ezetimibe, 1.14% ND-statins, 0.75% ND-statins plus ezetimibe, 0.09% PCSK9-i.

In LLT treated patients, the percentage of on-target patients was lowest (28.5%) in the very high risk category compared to the others. In Table 2, LLT use distribution at admission is reported according to CV risk classification (L, M, H, VH) and LDL-target achievement (on-, off-target). In VH category the proportion of on-target patients on monotherapy with statins or ezetimibe was significantly lower compared to the off-target group (1% vs. 2.3%, *P* = 0.026 for LI statins; 35.6% vs. 39.9%, *P* = 0.026 for MI statins; 35.5% vs. 40.9%, *P* = 0.005 for HI statins; 1.4% vs. 3.2%, *P* = 0.005 for ezetimibe). Conversely, the opposite was observed for combination therapies (1.6% vs. 0.4%, *P* = 0.001 for ND- statins plus ezetimibe; 2.2% vs. 1.1%, *P* = 0.028 for LI statins plus ezetimibe; 14.2% vs. 7.7%, *P* < 0.001 for MI statins plus ezetimibe; 7.4% vs. 3.3%, *P* < 0.001 for HI statins plus ezetimibe).

**Table 2:** Distribution of cholesterol targeting LLT use at admission for patients stratified by CV risk (low, moderate, high, very high) and LDL-target achievement (on-, off-target). Categorical variables are reported as numbers and relative percentages and *p*-values refer to Chi-square (χ²) test

	Low			Moderate			High			Very High		
	on-target	off-target	*p*-value	on-target	off-target	*p*-value	on-target	off-target	*p*-value	on-target	off-target	*p*-value
*N*	76	35		235	105		278	306		954	2396	
ND-statin (%)	0 ( 0.0)	0 ( 0.0)	*NA*	3 ( 1.3)	2 ( 1.9)	*1.000*	5 ( 1.8)	4 ( 1.3)	*0.885*	10 ( 1.0)	26 ( 1.1)	*1.000*
LI-statin (%)	2 ( 2.6)	1 ( 2.9)	*1.000*	9 ( 3.8)	7 ( 6.7)	*0.388*	8 ( 2.9)	16 ( 5.2)	*0.222*	10 ( 1.0)	55 ( 2.3)	***0.026***
MI-statin (%)	45 (59.2)	24 (68.6)	*0.463*	136 (57.9)	46 (43.8)	***0.022***	174 (62.6)	175 (57.2)	*0.213*	340 (35.6)	955 (39.9)	***0.026***
HI-statin (%)	16 (21.1)	8 (22.9)	*1.000*	47 (20.0)	29 (27.6)	*0.156*	49 (17.6)	67 (21.9)	*0.235*	339 (35.5)	980 (40.9)	***0.005***
ezetimibe (%)	3 ( 3.9)	2 ( 5.7)	*1.000*	7 ( 3.0)	7 ( 6.7)	*0.199*	4 ( 1.4)	18 ( 5.9)	***0.009***	13 ( 1.4)	76 ( 3.2)	***0.005***
PCSK9i (%)	0 ( 0.0)	0 ( 0.0)	*NA*	0 ( 0.0)	0 ( 0.0)	*NA*	0 ( 0.0)	0 ( 0.0)	*NA*	0 ( 0.0)	4 ( 0.2)	*0.479*
ND-statin + ezetimibe (%)	2 ( 2.6)	0 ( 0.0)	*0.841*	3 ( 1.3)	1 ( 1.0)	*1.000*	2 ( 0.7)	1 ( 0.3)	*0.934*	15 ( 1.6)	9 ( 0.4)	***0.001***
LI-statin + ezetimibe (%)	2 ( 2.6)	0 ( 0.0)	*0.841*	8 ( 3.4)	3 ( 2.9)	*1.000*	5 ( 1.8)	2 ( 0.7)	*0.374*	21 ( 2.2)	27 ( 1.1)	***0.028***
MI-statin + ezetimibe (%)	4 ( 5.3)	0 ( 0.0)	*0.404*	14 ( 6.0)	9 ( 8.6)	*0.514*	19 ( 6.8)	20 ( 6.5)	*1.000*	135 (14.2)	184 ( 7.7)	***< 0.001***
HI-statin + ezetimibe (%)	2 ( 2.6)	0 ( 0.0)	*0.841*	8 ( 3.4)	1 ( 1.0)	*0.350*	12 ( 4.3)	3 ( 1.0)	***0.022***	71 ( 7.4)	80 ( 3.3)	***< 0.001***

Abbreviations. HI-statin: high-intensity statin; LI-statin: low-intensity statin; MI-statin: medium-intensity statin; ND-statin: statin without data on treatment dosage; PCSK9i: proprotein convertase subtilisin/kexin type 9-inhibitors

## Discussion

This study presents real-world evidence on the distribution of risk classes, LDL-C target achievement and the use of LLT in a population of inpatients between the years 2021–2022. National and multicountry registries have previously reported LDL-C levels in primary and secondary care clinics [18], in specific patient categories, such as diabetes [6], secondary prevention [5] and in the high and very high-risk categories [7] (i.e., in selected populations already followed-up for their cardiovascular risk). To our knowledge, this is the first study which reports LDL-C levels and the percentage of target achievement in subjects from the general population at hospital admission (therefore in a condition of increased frailty).

A total of 109,234 hospitalizations were identified through an accurate extraction workflow, indicating a highly representative cohort of hospitalized individuals. Unfortunately, LDL-C values were unavailable for the majority of patients. This gap may be due to the large share of short hospital stays related to routine medical procedures or minor surgeries. In a small but non-negligible percentage of patients only a partial evaluation of lipid profile was performed (i.e. total cholesterol and triglycerides only), thereby precluding any assessment of LDL-C.

After further selection, 13,834 subjects with fasting LDL-C between 0 and 400 mg/dL which remained stable during hospitalization were identified. As reported, average age was 65.2 ± 16.5 years, many patients also had relevant comorbidities, such as diabetes, obesity and CKD, and almost one third had ASCVD. This cohort mirrors the general population because it includes subjects previously managed in other care settings, and no restrictive criteria were applied to the entire population examined, except for age > 18 years. The population was highly heterogeneous because it was drawn from all the hospital departments, but it specifically included many frail subjects. Indeed, in geriatric patients, frailty and higher hospitalization rates are closely linked [19]. Previous studies have documented that the increase in the number of comorbidities contributes to a decrease in the level of performance of complex daily living activities which in turn is associated with frailty [20]. In this study, hospitalization together with an elevated comorbidity burden defines a population of frail people.

Since this study examines an entire population and not subjects in follow-up at dyslipidemia centers [7, 18] a higher number of low-risk patients were included as well, likely admitted for the management and care of acute diseases without an impact on the cardiovascular system. Conversely, ASCVD prevalence was lower. For example, in the DA-VINCI study clinicians specifically enrolled patients, mainly in selected centers, and with a 1:1 ratio between primary and secondary prevention. ASCVD prevalence was non negligible in this cohort since “Cerebral Infarction due to thrombosis of unspecified cerebral artery” and “Coronary atherosclerosis of native coronary artery” were the most prevalent diagnoses after “Covid-19 pneumonia”. However, just under one third of subjects were admitted for the latter condition. The high percentage of Covid pneumonia is linked to the correspondence of this time interval with the pandemic and post-pandemic period and the central role assigned to this hospital in dealing with this emergency.

This study reveals that there are less on-target patients towards the higher CV risk categories, with only 21.4% of patients in the worst category reaching the target. However, there are a number of factors that must be considered in this analysis. First of all, the short interval from the publication of the 2019 ESC/EAS guidelines and the outbreak of Covid-19 pandemic. The latter has limited access to care for many subjects. Follow-up of many diseases has been neglected, thus preventing the application of the most recent guidelines. Secondly, a relevant contributing factor was the potential error in cardiovascular risk assessment, also described in other cohorts. Morieri et al. demonstrated that in type 2 diabetes patients, physician-based assessment of cardiovascular risk was incorrect in 34.7% of cases [21]. This population includes many multi-morbid elderly people, in which the risk is often conferred by the combination of pathologies and is therefore less straightforward than for ASCVD. Notably, 17.96% of non-ASCVD patients are in the highest risk category. Similarly, a study carried out in Korea showed that only 25.6% of patients with ASCVD and T2D achieved LDL-C target, but goal attainment was even worse in patients with target organ damage (TOD) associated with diabetes (15.7%) [22]. Additional reasons for this result include a higher prevalence of more severe diseases, potentially leading to an underestimation of cardiovascular risk and/or lower compliance to therapies, and statin intolerance. On the other hand, other more specific circumstances leading to overestimation of LDL-C target achievement should also be considered, e.g., severe sepsis, in which cholesterolemia can be reduced [23]. Frailty and comorbidity burden increase the risk of MACE [10, 12], therefore proper cardiovascular risk management is a priority in hospitalized patients. As this cohort mirrors the general population, these findings should serve as a warning to clinicians to prioritize achievement of target LDL-C in caring for hospitalized patients and will hopefully also encourage both general practitioners and specialists to intensify LLT.

This study also highlights the inadequate use of high-intensity statins and extremely low use of combination therapies in the general population and, therefore, the failure to reach target values even in subjects on LLT. Similarly, a cross-sectional observational study conducted in Portugal showed that more than half of LLT treated patients were off target [24]. According to ESC/EAS guidelines high-intensity statins must be prescribed up to the highest tolerated dose to reach the goals set for the specific risk level and, if the goal is not achieved, addition of ezetimibe is recommended. In addition, after drug-eluting stent implantation, combination therapy reduced MACE, statin discontinuation, and new-onset diabetes (NOD) compared to statins alone [25]. Increasing evidence is in favor of combination therapy as the standard of care for hypercholesterolemia treatment [26]. These data are in line with the latter findings as they show that patients in the VH category reached predefined LDL-C target more frequently with combination therapies than with monotherapies. Thus, addition of ezetimibe to any intensity statin seems to be a valid and successful option for many patients, compared to statin alone. Further, PCSK9-i and the recently available bempedoic acid could help to reach LDL-C goals in off-target patients already treated with statins/ezetimibe [27] without affecting the risk of NOD [28].

Further research is needed to assess the effects of baseline LDL at hospital admission on MACE, CV mortality and other health outcomes after hospital discharge.

### Strengths and limitations

This study has several strengths: standardized procedures for standard-of-care clinical data extraction were used and data were organized in a dedicated real-world repository (the Gemelli Dyslipidemia DataMart). To our knowledge, this is the first study to analyze LDL-C management in an entire population of inpatients with a considerable number of subjects (*n* = 13,834 pts). However, the study also has some limitations: probably data on LLT was not retrospectively retrievable for all patients, thus reducing the sample of treated patients. Moreover, information on time of LLT introduction was unavailable, as was post-discharge data. Finally, the prevalence of FH was obtained solely by exemption codes, since genetic tests were not available, thus its prevalence could be underestimated.

## Conclusions

This study presents the implementation of an accurate and automated multiple source RWE-based methodology to assess CV risk and LDL-C target achievement in hospitalized patients. Study results highlight relevant gaps in LDL-C target achievement in a non-selected population, generally frail, which included all adult patients admitted at the Fondazione Policlinico Gemelli Hospital between 1st January 2021 and 31st August 2022, with available LDL-C values during hospitalization. These gaps were higher in VH risk patients who were also characterized by an inadequate use of combination therapies and high intensity statins, which, when used, increased the probability of reaching therapeutic targets. These findings reveal a strong need for proper CV risk stratification of inpatients and for greater attention to LDL-C management and lipid lowering therapy implementation, as well as an increased personalization of care, by leveraging the newest treatments for the appropriate risk categories. These results must, in future, lead to efforts directed towards reducing this burden of risk on healthcare systems and hospitals in particular.

### Electronic supplementary material

Below is the link to the electronic supplementary material.

Supplementary Material 1

## Data Availability

No datasets were generated or analysed during the current study.

## Abbreviations

ASCVD: Atherosclerotic cardiovascular disease
CKD: Chronic kidney disease
CVD: Cardiovascular disease
DTT: Distance-to-target
DWH: Data warehouse
eGFR: Estimated-glomerular filtration rate
EHR: Extraction health records
ETL: Extract, transform, load
FH: Familial hypercholesterolemia
H: High
HDL-C: High-density lipoprotein-cholesterol
HI: High-intensity
HIS: Hospital information system
ICD9-CM: International Classification of Diseases, 9th revision - Clinical Modification
L: Low
LDL-C: Low-density lipoprotein-cholesterol
LI: Low-intensity
LIS: Laboratory information system
LLT: Lipid-lowering therapy
M: Moderate
MACE: Major adverse cardiovascular event
MI: Moderate-intensity
NOD: New onset diabetes
RWD: Real world data
RWE: Real world evidence
SCORE: Systematic COronary Risk Evaluation
TG: Triglyceride
uACR: Urinary albumin-to creatine ratio
VH: Very high
