# Supplementary material for: Real-world evidence evaluation of LDL-C in hospitalized patients: a population-based observational study in the timeframe 2021–2022
Source: Lipids Health Dis. 2024 Jul 24;23:224. doi: 10.1186/s12944-024-02221-x (PMC11267803; doi:10.1186/s12944-024-02221-x)
Supplement: Supplementary file 1 — Supplementary Material 1 [file 12944_2024_2221_MOESM1_ESM.docx]

**Supplementary Material**

**Title: Real-world evidence evaluation of LDL-C in hospitalized patients: a population-based observational study in the timeframe 2021-2022**

Umberto Capece^1,2&^, Chiara Iacomini^3&^, Teresa Mezza^1,2,4^, Alfredo Cesario^5,6^, Carlotta Masciocchi^3^, Stefano Patarnello^3^, Andrea Giaccari^1,2*^, Nicoletta Di Giorgi^3^

1 Centro Malattie Endocrine e Metaboliche, Dipartimento di Scienze Mediche e Chirurgiche, Fondazione Policlinico Universitario Agostino Gemelli IRCCS, 00168, Rome, Italy.

2 Dipartimento di Medicina e Chirurgia Traslazionale, Università Cattolica del Sacro Cuore, 00168, Rome, Italy.

3 Real World Data Facility, Gemelli Generator, Fondazione Policlinico Universitario Agostino Gemelli IRCCS, 00168, Rome, Italy

4 Pancreas Unit, CEMAD Centro Malattie dell’Apparato Digerente, Medicina Interna e Gastroenterologia, Fondazione Policlinico Universitario Agostino Gemelli IRCCS, 00168, Rome, Italy

5 Gemelli Digital Medicine & Health, Rome, Italy

6 Open Innovation Unit, Scientific Directorate, Fondazione Policlinico Universitario A. Gemelli IRCCS, 00168, Rome, Italy

^&^ These two authors contributed equally

***** Correspondence: andrea.giaccari@unicatt.it

**Table of contents**

[Table S1. Complete list of substances considered as artificial nutrition and/or interfering (non-lipid-lowering) drugs. 3](#_Toc168670511)

[Table S2. Data Dictionary: variables included in the Dyslipidaemia Data Mart. 6](#_Toc168670512)

[Table S3. Formulas used for the extraction of calculated variables. 7](#_Toc168670513)

[Table S4. Variable mapping to FPG Data Warehouse. 8](#_Toc168670514)

[Table S5. Availability assessment for multiple data sources considered for comorbidity status. 9](#_Toc168670515)

[Table S6. Anti-diabetic drugs. 10](#_Toc168670516)

[Table S7. Lipid-lowering therapies. 12](#_Toc168670517)

[Table S8. Clinical characteristics and risk factors by different LDL-C class according to 2019-ESC/EAS guidelines. 14](#_Toc168670518)

[Table S9. LDL-C levels and percentage of on/off-target patients in specific categories of patients: elderly (>= 80 years), critically ill patients, cancer patients and pregnant women. 15](#_Toc168670519)

Table S1. Complete list of substances considered as artificial nutrition and/or interfering (non-lipid-lowering) drugs.

| **Foods** | ALITRAQ VANIGLIA 6BUST 76G | oral solution |
| --- | --- | --- |
|  | CUBITAN CIOCCOLATO 200ML | oral solution |
|  | DIALYCARE VAN 237ML | oral solution |
|  | DIASON ALIM DIET 1000ML | oral solution |
|  | ENSURE TWOCAL BANANA 200ML | oral solution |
|  | FRESUBIN HEPA DRINK CAPPUCCINO | oral solution |
|  | GLUCERNA SR FRAGOLA 220ML | oral solution |
|  | GLUCERNA SR VANIGLIA 220ML | oral solution |
|  | IMPACT ORAL VANIGLIA 3X237ML | oral solution |
|  | JEVITY PLUS NEUTRO RTH 500ML | oral solution |
|  | MODULEN IBD LATTE POLVERE 400G | powder oral solution |
|  | NUTRIDRINK BANANA 4X200ML | oral solution |
|  | NUTRIDRINK CIOCCOLATO 4X200ML | oral solution |
|  | NUTRIDRINK VANIGLIA 4X200ML | oral solution |
|  | NUTRINI 200ML VF | oral solution |
|  | NUTRINI ENERGY PACK 500ML NP | oral solution |
|  | NUTRISON ADVANCED PROTISON 500 | oral solution |
|  | NUTRISON ALIM DIET SAC 1000ML | oral solution |
|  | NUTRISON ENERGY 1L | oral solution |
|  | NUTRISON MCT 1L | oral solution |
|  | NUTRISON MULTIFIBRE VT 500ML | oral solution |
|  | NUTRISON SOYA 1L | oral solution |
|  | NUTRISON SOYA 500ML | oral solution |
|  | OSMOLITE NEUTRO RTH 500ML | oral solution |
|  | OXEPA NEUTRO RTH 500ML | oral solution |
|  | PEPTISORB 500ML | oral solution |
|  | PROTIFAR POLVERE 225G | powder oral solution |
| **Glucose for compounds** | GLUCOSIO 10% | injectable solution |
|  | GLUCOSIO 5% | injectable solution |
| **Anhydrous glucose (dextrose)/sodium chloride** | GLUCOSIO SODIO CL II*500ML | injectable solution |
|  | GLUCOSIO SODIO CL II*500ML | injectable solution |
|  | GLUCOSIO SODIO CL II*500ML FL | infusion solution |
|  | GLUCOSIO SODIO CL II FKI*500ML | infusion solution |
| **Glucose (dextrose) monohydrate** | GLUCOSIO*10% 10F 10ML | injectable solution |
|  | GLUCOSIO*10% 1FL 500ML | injectable solution |
|  | GLUCOSIO*10% 20F 10ML | injectable solution |
|  | GLUCOSIO*10% 5F 10ML | injectable solution |
|  | GLUCOSIO*20% 1FL 500ML | injectable solution |
|  | GLUCOSIO*33% 10F 10ML | injectable solution |
|  | GLUCOSIO*33% 10F 10ML | injectable solution |
|  | GLUCOSIO*33% 1FL 500ML | injectable solution |
|  | GLUCOSIO*33% 1FL 500ML | infusion solution |
|  | GLUCOSIO*33% 5F 10ML | injectable solution |
|  | GLUCOSIO*50% 1FL 500ML | injectable solution |
|  | GLUCOSIO*50% 1FL 500ML | infusion solution |
|  | GLUCOSIO*50% 500ML | injectable solution |
|  | GLUCOSIO*50% SACCA 5000ML | infusion solution |
|  | GLUCOSIO*5% 10F 10ML | injectable solution |
|  | GLUCOSIO*5% 10FL 500ML | infusion solution |
|  | GLUCOSIO*5% 10 SACCHE 1000ML | infusion solution |
|  | GLUCOSIO*5% 1FL 250ML | injectable solution |
|  | GLUCOSIO*5% 1FL 500ML | injectable solution |
|  | GLUCOSIO*5% 20FL 250ML | infusion solution |
|  | GLUCOSIO*5% 20SACCHE 500ML | infusion solution |
|  | GLUCOSIO*5% 30SACCHE 250ML | infusion solution |
|  | GLUCOSIO*5% 50 SACCHE 100ML | infusion solution |
|  | GLUCOSIO*5% 75SACCHE 50ML | infusion solution |
|  | GLUCOSIO FKI*33% 1FL 500ML | infusion solution |
|  | GLUCOSIO FKI*5% 20FL 250ML | infusion solution |
|  | GLUCOSIO FKI*5% 20FL 500ML | infusion solution |
|  | GLUCOSIO GS*33% 10F 10ML | injectable solution |
|  | GLUCOSIO GS*5% 10F 10ML | injectable solution |
|  | GLUCOSIO SCLAVO*SCIR 150ML 75G | syrup |
| **Potassium chloride/glucose (dextrose) monohydrate** | POTASSIO CLOR 0,6% GLUC 5%*500 | injectable solution |
| **Glucose (dextrose) monohydrate/sodium chloride/calcium chloride dihydrate/magnesium chloride hexahydrate/sodium bicarbonate/sodium lactate** | PHYSIONEAL*40 GL 5SA 2LT 1,36% | peritoneal dialysis solution |
|  | PHYSIONEAL*40 GL 5SA 2LT 2,27% | peritoneal dialysis solution |
|  | PHYSIONEAL*40 GL 5SA 2LT 3,86% | peritoneal dialysis solution |
| **Glucose (dextrose) anhydrous/sodium acetate/sodium chloride/potassium chloride/magnesium chloride/calcium chloride/scetic acid** | ISOLYTE*INFUS 6SACC 2000ML | infusion solution |
| **Glucose (dextrose) monohydrate/polyamino acids/mineral salts/soybean oil** | KABIVEN*3SACCHE 2566ML BIOFINE | emulsion for infusion |
|  | KABIVEN*4SACCHE 2053ML BIOFINE | emulsion for infusion |
| **Glucose (dextrose) monohydrate/polyamino acids/purified soybean oil/purified olive oil/mineral salts** | OLICLINOMEL*N-6-900E 2000ML4SA | emulsion for infusion |
|  | OLICLINOMEL*N-6-900E 2500ML2SA | emulsion for infusion |
| **Amino acids/electrolytes/glucose (dextrose) anhydrous/lipids** | OLIMEL*N12E INF 6SACCHE 1000ML | emulsion for infusion |
|  | OLIMEL*N4E INF 2SACCHE 2500ML | emulsion for infusion |
|  | OLIMEL*N4E INF 4SACCHE 2000ML | emulsion for infusion |
|  | OLIMEL*N5E INF 2SACCHE 2500ML | emulsion for infusion |
|  | OLIMEL*N5E INF 4SACCHE 2000ML | emulsion for infusion |

Table S2. Data Dictionary: variables included in the Dyslipidaemia Data Mart.

| Category | Variable name | Time reference | Type |
| --- | --- | --- | --- |
| Demographic | Age; Gender; Patient ID | At admission | Structured |
| Hospitalization information | Patient ID; Admission ID; Reason for admission; Order of admission; Date/Time of admission; Date/Time of discharge; Diagnosis | At first admission during inclusion period | Structured |
| Laboratory exams | HDL-C; LDL-C; Total Cholesterol; Triglycerides | During hospitalization | Structured |
|  | Albuminuria; Creatinine; eGFR^S3^; Fasting Glucose; HbA1c; UACR | All before discharge | Structured |
| Diagnosis | Arteriopathy; ASCVD; CKD; Diabetes; Obesity; Myocardial infarction; Obesity | During hospitalization | Structured |
| Payment exemptions | ASCVD; CKD; Diabetes | All before discharge | Structured |
| Observations | BMI^S3^; DBP; Height; SBP; Weight | During hospitalization | Structured |
| Other comorbidities | Arteriopathy; Myocardial infarction | All before discharge | Unstructured  (Clinical diaries, clinical history, discharge letters) |
| Risk factors | Smoking Status | During hospitalization | Structured + Unstructured (Medical history) |
| Medications | Anti-diabetic drugs | All | Unstructured  (Medical history, clinical diaries, discharge letters) |
|  | Lipid-lowering therapies | Before and during hospitalization | Structured +  Unstructured  (Medical history, clinical diaries, discharge letters) |
| Nutritional | Foods, glucose-based nutritional products, glucose and lipid-based nutritional products | During hospitalization | Structured |

**Abbreviations.** ASCVD, atherosclerotic cardiovascular disease; BMI, body mass index; CKD, chronic kidney disease; DBP, diastolic blood pressure; eGFR, estimated glomerular filtration rate; HbA1c, glycated hemoglobin; HDL-C, high-density lipoprotein cholesterol; LDL-C, low-density lipoprotein cholesterol; SBP, systolic blood pressure; UACR, urinary albumin-to-creatinine ratio.

Table S3. Formulas used for the extraction of calculated variables.

| Variable name | Formula |
| --- | --- |
| Estimated glomerular filtration rate (eGFR), calculated using the CKD-EPI equation | $eGFR_{Cr}=142*{\min\left( \frac{S_{Cr}}{k},1 \right)}^{\alpha}*{\max\left( \frac{S_{Cr}}{k},1 \right)}^{-1.200}*{0.9938}^{Age}*1.012 (if female)$  where:  $S_{Cr}= standardized serum creatinine in mg/dL$  $k=0.7 \left( females \right) or 0.9 \left( males \right)$  $\alpha= - 0.241 \left( female \right) or-0.302 (male)$  $\min\left( \frac{S_{Cr}}{k},1 \right)is the minimum of\frac{S_{Cr}}{k} and 1.0$  $\max\left( \frac{S_{Cr}}{k},1 \right)is the maximum of\frac{S_{Cr}}{k}and 1.0$  $Age (years)$ |
| Body Mass Index (BMI) | $BMI (\frac{kg}{m^{2}})=\frac{weight\left( kg \right)}{\left( height\left( m \right) \right)^{2}}$ |

Table S4. Variable mapping to FPG Data Warehouse.

| **Comorbidity** | **Structured sources** | | | | | **Unstructured sources** | | **Rule for source integration** |
| --- | --- | --- | --- | --- | --- | --- | --- | --- |
|  | **ICD9-CM** | **Exemption code** | **Laboratory / observational parameters** | | | **Therapy** | |  |
|  |  |  | **Parameter** | **Threshold** | **Time range** | **Drugs/ ATC** | **Text source** |  |
| **Diabetes mellitus** | 250.* | 013  013.250 | HbA1c | ≥ 6.5% | before discharge date | 152 drugs / 51 ATC | hospitalizations clinical reports | OR |
|  |  |  | Fasting blood glucose | ≥ 126 mg/dL | between pre-hospitalization/admission and the 5th day from admission |  |  |  |
| **Chronic kidney disease** | 585.* | 023  023.585 | eGFR | <60 mL/min/1.73 m2 | before discharge date | / | / | OR |
|  |  |  | UACR | ≥ 30 mg/g | before discharge date |  |  |  |
| **Obesity** | 278.0* | / | BMI | ≥ 30 kg/m2 | between 6 months before and 30 days after admission | / | / | OR |
| **ASCVD** | 414.0 429.2  410.*  411.*  412  434.91  433.11 | 0C02.440  002.440 | / | / | / | / | / | OR |

**Abbreviations.** ASCVD, atherosclerotic cardiovascular disease; BMI, body mass index; eGFR, estimated glomerular filtration rate; HbA1c, glycated hemoglobin; HDL-C, high-density lipoprotein cholesterol; LDL-C, low-density lipoprotein cholesterol; UACR, urinary albumin-to-creatinine ratio.

Table S5. Availability assessment for multiple data sources considered for comorbidity status.

| **Comorbidity** | **Sources** | **Availability** | **Patients with comorbidity** |
| --- | --- | --- | --- |
| **Diabetes mellitus** | ICD9-CM | 13.834 (100%) | 1.689 (12.2%) |
|  | Waivers | 13.834 (100%) | 901 (6.5%) |
|  | Anti-diabetic therapy | 13.834 (100%) | N medicines ≥ 1 (excluding A10BA02), 2.023 (14.6%) |
|  | HbA1c | 5.894 (42.6%) | 1.911 (13.8%) |
|  | Fasting blood glucose | 12.621 (91.2%) | 2.568 (18.6%) |
| **Chronic kidney disease** | ICD9-CM | 13.834 (100%) | 676 (4.9%) |
|  | Waivers | 13.834 (100%) | 216 (1.6%) |
|  | eGFR | 13.822 (99.9%) | 4.863 (35.2%) |
|  | UACR | 454 (3.3%) | 178 (1.3%) |
| **Obesity** | ICD9-CM | 13.834 (100%) | 452 (3.3%) |
|  | BMI | 13.690 (99%) | 2.403 (17.4%) |
| **ASCVD** | ICD9-CM | 13.834 (100%) | 1.303 (9.4%) |
|  | Waivers | 13.834 (100%) | 4 (0.03%) |

**Abbreviations.** ASCVD, atherosclerotic cardiovascular disease; BMI, body mass index; eGFR, estimated glomerular filtration rate; HbA1c, glycated hemoglobin; HDL-C, high-density lipoprotein cholesterol; LDL-C, low-density lipoprotein cholesterol; UACR, urinary albumin-to-creatinine ratio.

Table S6. Anti-diabetic drugs.

| **ATC** | **Active ingredient** | **Active ingredient (IT)** | **Drug name** |
| --- | --- | --- | --- |
| A10AB01 | insulin (human) | insulina-umana | actrapid, humulin-r |
| A10AB04 | insulin lispro | insulina-lispro, insulina-lispro-insulina-lispro-protamina | humalog, humalog-junior-kwikpen, humalog-kwikpen, insulin-lispro-sanofi, humalog-mix-25, humalog-mix-25-kwikpen, humalog-mix-50-kwikpen |
| A10AB05 | insulin aspart | insulina-aspart | fiasp, novorapid-flexpen, novorapid-penfill, novorapid-pumpcart |
| A10AB06 | insulin glulisine | insulina-glulisina | apidra |
| A10AC01 | insulin human | insulina-umana, insulina-umana-isofano | insuman-rapid, humulin-i, protaphane |
| A10AD01 | insulin human | insulina-isofano-bifasica-preparazione-iniettabile, insulina-umana-insulina-umana-isofano | humulin-3070, actraphane-30 |
| A10AD05 | insulin aspart | insulina-aspart, insulina-aspart-insulina-aspart-protamina | insulina-aspart-sanofi, novomix-30-flexpen, novomix-50-flexpen, novomix-70-flexpen |
| A10AE04 | insulin glargine | insulina-glargine | abasaglar, lantus, lantus-solostar, toujeo |
| A10AE05 | insulin detemir | insulina-detemir | levemir |
| A10AE06 | insulin degludec | insulina-degludec | tresiba |
| A10AE54 | insulin glargine and lixisenatide | insulina-glargine-lixisenatide | suliqua |
| A10AE56 | insulin degludec and liraglutide | insulina-degludec-liraglutide | xultophy |
| A10BA02 | metformin | metformina-cloridrato, metformina | glucophage, glucophage-unidie, keymet, metfonorm, metforal, metforalmille, metformina-aurobindo, metformina-doc, metformina-eg, metformina-eg-stada-group, metformina-hexal, metformina-hexal-ag, metformina-mylan-generics, metformina-teva, metformina-teva-italia, slowmet, zuglimet, metformina-almus, metformina-pensa |
| A10BB01 | glibenclamide | glibenclamide | daonil, gliben, gliboral |
| A10BB07 | glipizide | glipizide | minidiab |
| A10BB08 | gliquidone | gliquidone | glurenor |
| A10BB09 | gliclazide | gliclazide | diabrezide, diamicron, dramion, gleukos, gliclazide-doc-generici, gliclazide-doc-generics, gliclazide-eg, gliclazide-eg-stada, gliclazide-krka, gliclazide-molteni, gliclazide-mylan-generics, gliclazide-pensa, gliclazide-teva, gliclazide-teva-italia, gliclazide-zentiva, gliclazide-zentiva-lab |
| A10BB12 | glimepiride | glimepiride | amaryl, diamel, glimepiride-accord-healthcare, glimepiride-aurobindo, glimepiride-eg, glimepiride-sandoz-gmbh, solosa |
| A10BD02 | metformin and sulfonylureas | metformina-glibenclamide, metformina-sulfonamidi | diaglimet, glibomet, gliconorm, glicorest, suguan-m |
| A10BD05 | metformin and pioglitazone | pioglitazone-metformina | competact, glubrava, pioglitazone-e-metformina-aristo, pioglitazone-e-metformina-docgen, pioglitazone-e-metformina-eg |
| A10BD06 | glimepiride and pioglitazone | pioglitazone-glimepiride | tandemact |
| A10BD07 | metformin and sitagliptin | sitagliptin-metformina | efficib, janumet, velmetia |
| A10BD08 | metformin and vildagliptin | vildagliptin-metformina | eucreas |
| A10BD09 | pioglitazone and alogliptin | pioglitazone-alogliptin | incresync |
| A10BD10 | metformin and saxagliptin | saxagliptin-metformina | komboglyze |
| A10BD11 | metformin and linagliptin | metformina-linagliptin | jentadueto |
| A10BD13 | metformin and alogliptin | metformina-alogliptin | vipdomet |
| A10BD15 | metformin and dapagliflozin | metformina-dapagliflozin | xigduo |
| A10BD16 | metformin and canagliflozin | metformina-canagliflozin | vokanamet |
| A10BD19 | linagliptin and empagliflozin | linagliptin-empagliflozin | glyxambi |
| A10BD20 | metformin and empagliflozin | metformina-empagliflozin | synjardy |
| A10BD21 | saxagliptin and dapagliflozin | saxagliptin-dapagliflozin | qtern |
| A10BD23 | metformin and ertugliflozin | ertugliflozin-metformina-cloridrato | segluromet |
| A10BD24 | sitagliptin and ertugliflozin | ertugliflozin-sitagliptin | steglujan |
| A10BF01 | acarbose | acarbose | acarbosio-doc-generici, acarbosio-tecnigen, acarphage, glucobay |
| A10BG03 | pioglitazone | pioglitazone-cloridrato | actos, pioglitazone-accord, pioglitazone-actavis, pioglitazone-aurobindo, pioglitazone-doc-generici, pioglitazone-mylan, pioglitazone-sandoz, pioglitazone-tecnigen |
| A10BH01 | sitagliptin | sitagliptin, sitagliptin-fosfato-monoidrato | sitagliptin-doc, sitagliptin-eg, sitagliptin-hcs, sitagliptin-mylan, januvia, tesavel, xelevia |
| A10BH02 | vildagliptin | vildagliptin | galvus, vildagliptin-accord, vildagliptin-eg |
| A10BH03 | saxagliptin | saxagliptin | onglyza |
| A10BH04 | alogliptin | alogliptin | vipidia |
| A10BH05 | linagliptin | linagliptin | trajenta |
| A10BJ01 | exenatide | exenatide | bydureon, byetta |
| A10BJ03 | lixisenatide | lixisenatide | lyxumia |
| A10BJ05 | dulaglutide | dulaglutide | trulicity |
| A10BJ06 | semaglutide | semaglutide | ozempic, rybelsus |
| A10BK01 | dapagliflozin | dapagliflozin-propanediolo-monoidrato | forxiga |
| A10BK02 | canagliflozin | canagliflozin-emiidrato | invokana |
| A10BK03 | empagliflozin | empagliflozin | jardiance |
| A10BK04 | ertugliflozin | ertugliflozin | steglatro |
| A10BX02 | repaglinide | repaglinide | glicam, novonorm, repaglinide-accord, repaglinide-aurobindo, repaglinide-doc-generici, repaglinide-eg, repaglinide-krka, repaglinide-mylan-generics, repaglinide-sandoz, repaglinide-teva, repaglinide-zentiva, theroflan |
| A10BX07 | liraglutide | liraglutide | victoza |

Table S7. Lipid-lowering therapies.

| **ATC** | **Active ingredient** | **Active ingredient (IT)** | **Drug name** | **Target** |
| --- | --- | --- | --- | --- |
| C10AA01 | simvastatin | simvastatina | alpheus, krustat, lipenil, liponorm, medipo, omistat, rosim, setorilin, simbatrix, simvastatina-abc, simvastatina-accord, simvastatina-almus, simvastatina-alter, simvastatina-aristo, simvastatina-aurobindo, simvastatina-doc, simvastatina-eg, simvastatina-hexal, simvastatina-krka, simvastatina-mylan-generics, simvastatina-sandoz, simvastatina-sun, simvastatina-tecnigen, simvastatina-teva-italia, simvastatina-zentiva, sincol, sinvacor, sinvalip, sinvat, sivastin, vastgen, vastin, xipocol, zocor | Cholesterol |
| C10AA02 | lovastatin | lovastatina | lovastatina-doc, lovastatina-eg, lovastatina-mylan-pharma, lovastatina-teva, lovinacor, rextat, tavacor | Cholesterol |
| C10AA03 | pravastatin | pravastatina-sale-sodico, pravastatina | aplactin, langiprav, prasterol, pravaselect, pravastatina-almus, pravastatina-alter, pravastatina-aurobindo, pravastatina-doc-generici, pravastatina-eg, pravastatina-mylan, pravastatina-ratiopharm, pravastatina-sandoz-gmbh, pravastatina-sun, pravastatina-zentiva, setac, vasticor | Cholesterol |
| C10AA04 | fluvastatin | fluvastatina-sale-sodico | fluvastatina-doc-generici, fluvastatina-eg, fluvastatina-teva, fluvastatina-zentiva, lescol, lipaxan | Cholesterol |
| C10AA05 | atorvastatin | atorvastatina-sale-di-calcio, atorvastatina | arkas, atoris, atorvastatina-abc, atorvastatina-accord, atorvastatina-almus-pharma, atorvastatina-alter, atorvastatina-aristo-pharma, atorvastatina-aurobindo, atorvastatina-docgen, atorvastatina-eg, atorvastatina-firma, atorvastatina-git, atorvastatina-krka, atorvastatina-mylan-generics-italia, atorvastatina-nisura, atorvastatina-pensa, atorvastatina-sandoz-gmbh, atorvastatina-sun, atorvastatina-tecnigen, atorvastatina-teva-italia, atorvastatina-viatris, atorvastatina-zentiva, kolester, melemib, omegastatin, sopavi, taxat, torvacol, torvast, totalip, tovanira, vastat, xarator | Cholesterol |
| C10AA07 | rosuvastatin | rosuvastatina, rosuvastatina-sale-di-calcio | borghes, colcardiol, colfri, crativ, crestor, dilivas, exorta, koleros, lipidover, miastina, provisacor, rosastin, rosuvastatina-abc, rosuvastatina-abc-farmaceutici, rosuvastatina-adamed, rosuvastatina-adamed-pharma, rosuvastatina-almus, rosuvastatina-alter, rosuvastatina-aristo, rosuvastatina-aurobindo, rosuvastatina-doc-generici, rosuvastatina-eg, rosuvastatina-hcs, rosuvastatina-mylan, rosuvastatina-pensa, rosuvastatina-sandoz, rosuvastatina-sun, rosuvastatina-tecnigen, rosuvastatina-teva, rosuvastatina-zentiva, simestat, snaptag, staros, tasavos, tekvas | Cholesterol |
| C10AA08 | pitavastatin | pitavastatina-calcica | livazo | Cholesterol |
| C10AB02 | bezafibrate | bezafibrato | bezalip | Triglycerides |
| C10AB04 | gemfibrozil | gemfibrozil | gemfibrozil-doc-generici, gemfibrozil-eg, genlip, lopid | Triglycerides |
| C10AB05 | fenofibrate | fenofibrato | fenofibrato-doc-generici, fenofibrato-eg, fulcro, fulcrosupra, liperial, lipofene, lipsin | Triglycerides |
| C10AD06 | acipimox | acipimox | olbetam | Triglycerides |
| C10AX06 | omega-3-triglycerides incl. other esters and acids | acidi-omega-3-esteri-etilici-90, omega-polienoici, icosapent-etile | agemo, blumeg, esapent, eskim, olevia, omega-3-alfasigma, omega-3-bouty, omega-3-doc, omega-3-doc-generici, omega-3-eg, omega-3-mylan, omega-3-pensa, omega-3-sandoz, omega-3-teva-italia, omega-3-zentiva, seacor, trecor, tribok, trioreg, triscudil, vazkepa | Triglycerides |
| C10AX09 | ezetimibe | ezetimibe | absorcol, ezelip, ezetimibe-accord, ezetimibe-almus, ezetimibe-alter, ezetimibe-aristo, ezetimibe-aurobindo, ezetimibe-eg, ezetimibe-krka, ezetimibe-mylan, ezetimibe-pensa, ezetimibe-tecnigen, ezetimibe-teva, ezetimibe-zentiva, ezetrol, zetia | Cholesterol |
| C10AX12 | lomitapide | lomitapide | lojuxta | Triglycerides |
| C10AX13 | evolocumab | evolocumab | repatha | Cholesterol |
| C10AX14 | alirocumab | alirocumab | praluent | Cholesterol |
| C10AX16 | inclisiran | inclisiran | leqvio | Cholesterol |
| C10AX18 | volanesorsen | volanesorsen | waylivra | Triglycerides |
| C10BA02 | simvastatin and ezetimibe | ezetimibe-simvastatina | amisitela, ezetimibe-e-simvastatina-alter, ezetimibe-e-simvastatina-aurobindo, ezetimibe-e-simvastatina-doc-generici, ezetimibe-e-simvastatina-eg, ezetimibe-e-simvastatina-krka, ezetimibe-e-simvastatina-mylan, ezetimibe-e-simvastatina-sandoz, ezetimibe-e-simvastatina-tecnigen, ezetimibe-e-simvastatina-teva-b-v, goltor, inegy, staticol, vytorin, zestan, zevistat | Cholesterol |
| C10BA04 | simvastatin and fenofibrate | simvastatina-fenofibrato | cholib | Cholesterol |
| C10BA05 | atorvastatin and ezetimibe | ezetimibe-atorvastatina | ancilleg, ezetimibe-e-atorvastatina-doc, ezetimibe-e-atorvastatina-eg, ezevast, kexrolt, orvatez, tovastibe | Cholesterol |
| C10BA06 | rosuvastatin and ezetimibe | rosuvastatina-ezetimibe | aurozeb, cholecomb, compuna, ezateros, maoris, quiloga, rosetem, rosumibe, rosuvastatina-e-ezetimibe-doc, rosuvastatina-e-ezetimibe-teva, rozetimad | Cholesterol |
| C10BX05 | rosuvastatin and acetylsalicylic acid | rosuvastatina-acido-acetilsalicilico | asadrox, rosuasa | Cholesterol |
| C10BX09 | rosuvastatin and amlodipine | rosuvastatina-amlodipina | coaredam, rosulod, rozamlad, suvamod | Cholesterol |
| C10BX11 | atorvastatin, amlodipine and perindopril | atorvastatina-perindopril-amlodipina | triveram | Cholesterol |
| C10BX18 | atorvastatin, amlodipine and ramipril | ramipril-amlodipina-atorvastatina | trinocard | Cholesterol |

Table S8. Clinical characteristics and risk factors by different LDL-C class according to 2019-ESC/EAS guidelines.

|  | **All** | **<55mg/dL** | **55-70mg/dL** | **70-100mg/dL** | **>=100mg/dL** | **AOV/Chi-square test** |
| --- | --- | --- | --- | --- | --- | --- |
| **N** | **13,834** | **2,253 (16.3%)** | **2,264 (16.3 %)** | **4,848 (35%)** | **4,469 (32.3%)** |  |
| **Age (yrs)** | **65.2 ± 16.5** | 69.3 ± 15.2 | 67.3 ± 16.7 | 64.6 ± 17.3 | 62.6 ± 15.7 | *1.611e-64* |
| **Males** | **7908 (57.2%)** | 1480 (65.7%) | 1371 (60.6%) | 2751 (56.7%) | 2306 (51.6%) | *6.160e-29* |
| **Lipid profile** |  |  |  |  |  |  |
| **LDL-C (mg/dL)** | **87.4 ± 34.6** | **42.2 ± 9.3** | **62.4 ± 4.3** | **84.2 ± 8.7** | **126.1 ± 26.8** | ***0*** |
| **DTT (mg/dL)** | **12.4 ± 39.4** | -24.4 ± 21.5 | -8.4 ± 22.9 | 7.7 ± 26.1 | 47.3 ± 37.8 | *0* |
| **DTT %** | **25.7 ± 59.8** | -32.6 ± 20.2 | -4.5 ± 24.2 | 20.8 ± 36.1 | 76.6 ± 66.2 | *0* |
| **Total Cholesterol (mg/dL)** | **150.5 ± 42.8** | 98.1 ± 19.7 | 122 ± 16.5 | 147.1 ± 19 | 195.1 ± 33.9 | *0* |
| **Triglycerides (mg/dL)** | **123.2 ± 58.9** | 119.8 ± 61.6 | 116 ± 54.9 | 117.9 ± 53.6 | 134.3 ± 63.4 | *2.636e-52* |
| **HDL-C (mg/dL)** | **39 ± 15.2** | 31 ± 13.6 | 36.1 ± 13.9 | 39.8 ± 14.6 | 43.7 ± 15.4 | *3.150e-257* |
| **Risk Factors** |  |  |  |  |  |  |
| **BMI (kg/m2)** | **26.3 ± 5.7** | 26.3 ± 5.7 | 26.4 ± 6.2 | 26.1 ± 5.2 | 26.4 ± 6 | *0.0332* |
| **BP systolic (mmHg)** | **122.7 ± 12.9** | 120.9 ± 12.6 | 122.5 ± 13 | 122.8 ± 12.5 | 123.5 ± 13.2 | *1.316e-06* |
| **BP diastolic (mmHg)** | **72 ± 6.6** | 70.1 ± 5.8 | 71.5 ± 6.4 | 72.1 ± 6.4 | 73 ± 7 | *8.449e-60* |
| **Current Smokers** | **1504 (10.9%)** | 193 (8.6%) | 231 (10.2%) | 544 (11.2%) | 536 (12%) | *4.853e-08* |
| **Comorbidities** |  |  |  |  |  |  |
| **Diabetes mellitus** | **4218 (30.5%)** | 1044 (46.3%) | 825 (36.4%) | 1334 (27.5%) | 1015 (22.7%) | *8.852e-98* |
| **CKD** | **4937 (35.7%)** | 1191 (52.9%) | 988 (43.6%) | 1585 (32.7%) | 1173 (26.2%) | *1.137e-117* |
| **Obesity** | **2471 (17.9%)** | 399 (17.7%) | 434 (19.2%) | 820 (16.9%) | 818 (18.3%) | *0.1006* |
| No Comorbidities (among diabetes, CKD, obesity) | **5684 (41.1%)** | 548 (24.3%) | 762 (33.7%) | 2133 (44%) | 2241 (50.1%) | *4.483e-104* |
| Single Comorbidity | **5172 (37.4%)** | 910 (40.4%) | 874 (38.6%) | 1831 (37.8%) | 1557 (34.8%) | *4.442e-05* |
| 2 Comorbidities | **2480 (17.9%)** | 661 (29.3%) | 511 (22.6%) | 744 (15.3%) | 564 (12.6%) | *2.104e-73* |
| All 3 Comorbidities | **498 (3.6%)** | 134 (5.9%) | 117 (5.2%) | 140 (2.9%) | 107 (2.4%) | *9.908e-17* |
| **ASCVD** | **4401 (31.8%)** | 1048 (46.5%) | 848 (37.5%) | 1358 (28%) | 1147 (25.7%) | *6309e-65* |

**Abbreviations.** AOV, one-way ANOVA test; ASCVD, atherosclerotic cardiovascular disease; BMI, body mass index; BP, blood pressure; CKD, chronic kidney disease; HDL-C, high-density lipoprotein cholesterol; LDL-C, low-density lipoprotein cholesterol.

Table S9. LDL-C levels and percentage of on/off-target patients in specific categories : (A) patients with diabetes, chronic kidney disease (CKD), obesity and hypertension; (B) elderly (>= 80 years), critically ill patients, cancer patients and pregnant women; (C) current smokers.

|  |  | **Diabetes** | | | **CKD** | | | **Obesity** | | | **Hypertension** | | |
| --- | --- | --- | --- | --- | --- | --- | --- | --- | --- | --- | --- | --- | --- |
|  | **Overall** | **0** | **1** | ***p*** | **0** | **1** | ***p*** | **0** | **1** | ***p*** | **0** | **1** | ***p*** |
| **n** | **13834** | 9616 | **4218** |  | 8897 | **4937** |  | 11363 | **2471** |  | 9299 | **2873** |  |
| **LDL-C (mg/dL)** | **87.4 ± 34.6** | 90.92 (34.05) | **79.24 (34.55)** | *<0.001* | 92.05 (34.40) | **78.90 (33.40)** | *<0.001* | 87.21 (34.56) | **88.01 (34.90)** | *0.301* | 86.62 (35.06) | **87.99 (33.22)** | *0.064* |
| **off-target (%)** | **8361 (61.6)** | 5505 (58.7) | **2856 (67.9)** | *<0.001* | 4850 (56.1) | **3511 (71.2)** | *<0.001* | 6795 (61.0) | **1566 (63.9)** | *0.009* | 5518 (60.2) | **2000 (70.9)** | *<0.001* |
| **on-target (%)** | **5223 (38.4)** | 3870 (41.3) | **1353 (32.1)** |  | 3802 (43.9) | **1421 (28.8)** |  | 4338 (39.0) | **885 (36.1)** |  | 3644 (39.8) | **819 (29.1)** |  |

(A)

(B)

|  |  | **Elderly (>= 80 yrs)** | | | **Intensive care units** | | | **Cancer** | | | **Pregnancy** | | |
| --- | --- | --- | --- | --- | --- | --- | --- | --- | --- | --- | --- | --- | --- |
|  | **Overall** | **0** | **1** | ***p*** | **0** | **1** | ***p*** | **0** | **1** | ***p*** | **0** | **1** | ***p*** |
| **n** | **13834** | 10889 | **2945** |  | 13174 | **660** |  | 11778 | **2056** |  | 13757 | **77** |  |
| **LDL-C (mg/dL)** | **87.4 ± 34.6** | 89.4 ± 35.0 | **79.9 ± 32.0** | *<0.001* | 87.6 ± 34.7 | **82.1 ± 32.9** | *<0.001* | 87.3 ± 33.3 | **87.9 ± 41.3** | *0.436* | 87.2 ± 34.5 | **112.9 ± 37.6** | *<0.001* |
| **off-target (%)** | **8361 (61.6)** | 6189 (58.1) | **2172 (74.3)** | *<0.001* | 8023 (61.9) | **338 (53.9)** | *<0.001* | 7165 (62.1) | **1196 (58.5)** | *0.002* | 8315 (61.6) | **46 (59.7)** | *0.834* |
| **on-target (%)** | **5223 (38.4)** | 4470 (41.9) | **753 (25.7)** |  | 4934 (38.1) | **289 (46.1)** |  | 4375 (37.9) | **848 (41.5)** |  | 5192 (38.4) | **31 (40.3)** |  |

(C)

|  |  | **Current Smokers** | | |
| --- | --- | --- | --- | --- |
|  | **Overall** | **0** | **1** | ***p*** |
|  |  |  |  |  |
| **n** | **13834** | 3669 | **1504** |  |
| **LDL-C (mg/dL)** | **87.4 ± 34.6** | 86.30 (36.09) | **91.30 (35.45)** | *<0.001* |
| **off-target (%)** | **8361 (61.6)** | 2299 (63.0) | **1028 (68.8)** | *<0.001* |
| **on-target (%)** | **5223 (38.4)** | 1350 (37.0) | **467 (31.2)** |  |

0 is for the subjects that do not belong to the subgroup, 1 is for those that belong to the subgroup, *p* is for comparison between subgroups
